# Effectiveness of regular weighing, weight target setting and feedback by community midwives within routine antenatal care in preventing excessive gestational weight gain: randomised controlled trial

**DOI:** 10.1186/s40608-016-0086-4

**Published:** 2016-02-05

**Authors:** Amanda J. Daley, K. Jolly, S. A. Jebb, A. K. Roalfe, L. Mackillop, A. L. Lewis, S. Clifford, S. Kenyon, C. MacArthur, P. Aveyard

**Affiliations:** 1 Primary Care Clinical Sciences, School of Health and Population Sciences, College of Medical and Dental Sciences, University of Birmingham, Birmingham, B15 2TT UK; 2 Public Health, Epidemiology and Biostatistics, School of Health and Population Sciences, University of Birmingham, Birmingham, B15 2TT UK; 3 Nuffield Department of Primary Care Health Sciences, University of Oxford, Radcliffe Observatory Quarter, Woodstock Road, Oxford, OX2 6GG UK; 4 Women’s Centre, John Radcliffe Hospital, Headley Way, Oxford, OX3 9DU UK; 5 School of Social and Community Medicine, University of Bristol, Canynge Hall, 39 Whatley Road, Bristol, BS8 2PS UK

**Keywords:** Pregnancy weight, Self monitoring, Midwives, Weighing

## Abstract

**Background:**

Many pregnant women gain excess weight during pregnancy which increases the health risks to the mother and her baby. Interventions to prevent excess weight gain need to be given to the whole population to prevent excess weight gain. The aim of this study was to assess the effectiveness of a simple and brief intervention embedded withinroutine antenatal care to prevent excessive gestation weight gain.

**Methods:**

Six hundred and ten pregnant women (between 10-14 weeks gestation), aged ≥18 years with a body mass index (BMI) ≥18.5 kg/m2, planned to receive community midwife led care or shared care at the time of recruitment are eligible to take part in the study. Women will be recruited from four maternity centres in England. Community midwives complete a short training module before delivering the intervention. In the intervention, midwives weigh women, set maximum weight limits for weight gain at each antenatal appointment and ask women to monitor their weight at home. Themaximum weight limit is adjusted by the midwife at each antenatal appointment if women have exceeded their maximum weight gain limit set at their previous appointment. The intervention will be compared with usual antenatal care. The primary outcome is the proportion of women per group who exceed the Institute of Medicine guidelines for gestational weight gain at 38 weeks of pregnancy according to their early pregnancy BMI category.

**Discussion:**

The proposed trial will test a brief intervention comprising regular weighing, target setting and monitoring ofweight during pregnancy that can be delivered at scale as part of routine antenatal care. Using the professional expertise of community midwives, but without specialist training in weight management, the intervention will incur minimal additionalhealthcare costs, and if effective at reducing excess weight gain, is likely to be very cost effective.

**Trial registration:**

Current controlled trials ISRCTN67427351. Date assigned 29/10/2014.

## Background

Pregnancy is a critical risk period for the development of later obesity [1]. Average weight gain in pregnancy has increased dramatically in the last two decades and this is true for women across body mass index (BMI) categories [2, 3]. Most pregnant women in developed and developing countries exceed current USA Institute of Medicine (IOM) or similar guidance [4–7]. The IOM recommend that women with a healthy, overweight and obese pre pregnancy BMI should gain between 11.5–16 kg, 7–11.5 kg and 5–9 kg respectively. Although some countries monitor pregnant women to assess whether they are gaining appropriate weight, many do not, including the UK. Excessive gestational weight gain is associated with several adverse maternal and infant outcomes that could be prevented with better weight control, such as gestational diabetes, pre-eclampsia, delivery complications, caesarean sections, macrosomia and stillbirth [1, 8, 9]. Excess gestational weight gain is associated with postnatal weight retention up to 10–15 years after pregnancy in all BMI categories of women [10–14]. The weight women gain during pregnancy but fail to lose after pregnancy leads to incremental gains across successive pregnancies [12]. In addition, studies report an association between high maternal weight gain during pregnancy and increased adiposity and morbidity in children [15, 16].

Women often feel that gaining weight during pregnancy is inevitable and weight control is less important than at other life stages [17–19]. Studies have reported a lack of awareness amongst pregnant women about the risks of excessive gestational weight gain and many women reduce their physical activity and consume a more liberal diet when pregnant [20, 21]. There have been calls for weight management to be formally integrated into routine antenatal care in the UK and other countries but there is a paucity of evidence of effective interventions to prevent excessive gestational weight gain. Studies have reported that pregnant women feel health professionals should support and guide them about weight gain during pregnancy and that midwives are the most appropriate people to do so [22]. Community midwives have regular contact with women throughout pregnancy, providing multiple opportunities for them to intervene and this is consistent with their role to promote good outcomes for mother and baby. Yet weight management is not seen as a priority, nor is weight routinely monitored in the UK or many other countries [23] Studies show that women believe that if gestational weight gain was important their midwife would have discussed it with them [17], which may lead many pregnant women to conclude it is not a health risk to them or their baby if their weight is not discussed and/or monitored. Most randomised controlled trials (RCTs) to date have focussed on specialist interventions for obese women who are pregnant. This is important, but the majority of women who become pregnant are healthy or overweight, not obese, yet pregnancy may be the time when weight control slips and preventative interventions are needed for these women to minimise their long-term health risks and potential adverse effects on their baby.

### Evidence

Numerous recent systematic reviews [24–37] have assessed the effectiveness of weight management interventions during pregnancy. One of these [24] reviews showed an overall reduction in weight gain −0.97 kg in those randomised to weight management programmes compared with controls. For dietary based interventions the effect was larger at −3.36 kg. Analysis showed that dietary interventions resulted in significant reductions in preeclampsia, dystocia, gestational hypertension and pre term birth. Whilst the many systematic reviews to date have all had different inclusion and exclusion criteria, *without exception*, they have concluded there are insufficient high quality trials to judge the effectiveness of interventions to promote healthy gestational weight gain, with several reviews highlighting this as a particular omission in women who are not already obese. Moreover, trials to date have evaluated very intensive and costly interventions in obese pregnant women and there is a paucity of trials focused on preventing excessive gestational weight gain in all pregnant women.

### Previous relevant trials

A feasibility trial conducted in Finland provided evidence that involving midwives in delivery of weight control interventions is feasible [38]. It embedded an intensive diet and physical activity counselling intervention delivered by public health nurses (similar to community midwives) to low risk pregnant women. The intervention was found feasible to implement; the average participation rate of eligible women was 77 % and drop out was low at 15 %. An RCT enrolled 236 pregnant women of any BMI [39]. It evaluated an intervention where women were asked by a medical student researcher at 12 weeks gestation to weigh themselves and to plot their weight on a chart during pregnancy with no support thereafter, providing additional information that this type of intervention is feasible to implement. Compared to usual care however the intervention was not effective in preventing excessive weight gain. This could be because community midwives were not involved in reinforcing this message during pregnancy or checking the woman’s chart and providing feedback. Nor were key components of self-regulation such as goal setting and feedback included.

There have been trials that have demonstrated effective interventions that prevented excessive weight gain, but they have not been embedded in routine care. The recent FeLIPO trial (*n* = 250) conducted in Germany assessed the efficacy of an intervention to prevent excessive weight gain in women with a BMI ≥18.5 kg [40]. The intervention consisted of two individually delivered counselling sessions led by specialist staff focussing on diet, physical activity and weight monitoring lasting 60 and 30 min respectively and delivered at 20 and 30 weeks of pregnancy. The intervention led to a substantially lower proportion of women exceeding the IOM guidelines for gestational weight gain compared with the control group (38 % versus 60 %). An RCT conducted in the USA randomised women (*n* = 120) to usual care or a multi component behavioural intervention involving consultations with a researcher and dietician about healthy lifestyle behaviours, mailed prompts, encouragement of self monitoring of physical activity and energy intake, free weighing scales to encourage regular self monitoring and personalised feedback on weight gain [41]. Compared with standard care the intervention decreased the proportion of women of baseline healthy BMI who exceeded the IOM guidelines for gestational weight gain (40.2 % versus 52.1 %). These studies show that interventions can be effective in reducing excess weight gain among a general sample of pregnant women, but they require specialist staff and are more intensive and expensive than could be offered as part of routine antenatal care for all pregnant women. For primary and secondary prevention, low intensity and cost efficient interventions that can be given to all pregnant women are required; one approach that would meet these criteria is regular weighing, target setting and monitoring of weight during pregnancy delivered using the existing professional expertise of community midwives, rather than specialists in weight management.

### Target setting for healthy weight gain, regular weighing and feedback

There has been growing interest in the possibility of routine weighing of women during pregnancy in the UK but the National Institute for Health and Care Excellence (NICE) [42] concluded that the evidence base was insufficient to make recommendations, reinforcing the need for new research now. The potential efficacy of regular weighing and target setting (either by the individual or someone else, such as a health professional) is based on the principles of self regulation theory [43, 44]^.^ Self regulation has been described as a process that has three distinct stages; self monitoring, self evaluation and self reinforcement. Self monitoring is a method of systematic self observation, periodic measurement and recording of target behaviours with the goal of increasing self awareness. The awareness fostered during self monitoring is considered an essential initial step in promoting and sustaining behaviour change. Our own work in non pregnant populations and other studies [45–47] have showed that regular weighing can facilitate weight control; similar interventions during pregnancy, a time of dynamic weight change, may also have merit. However, even if regular weighing helps women control their weight during pregnancy, concerns have been expressed that it may have negative psychological consequences and that feedback about body size may result in anxiety, or lead to the adoption of unhealthy weight control practices during pregnancy. In non pregnant women however there is no evidence to support these concerns [48, 49].

### Feasibility work: Preventing Obesity in Pregnancy Study (POPS)

**Fig. 1:**
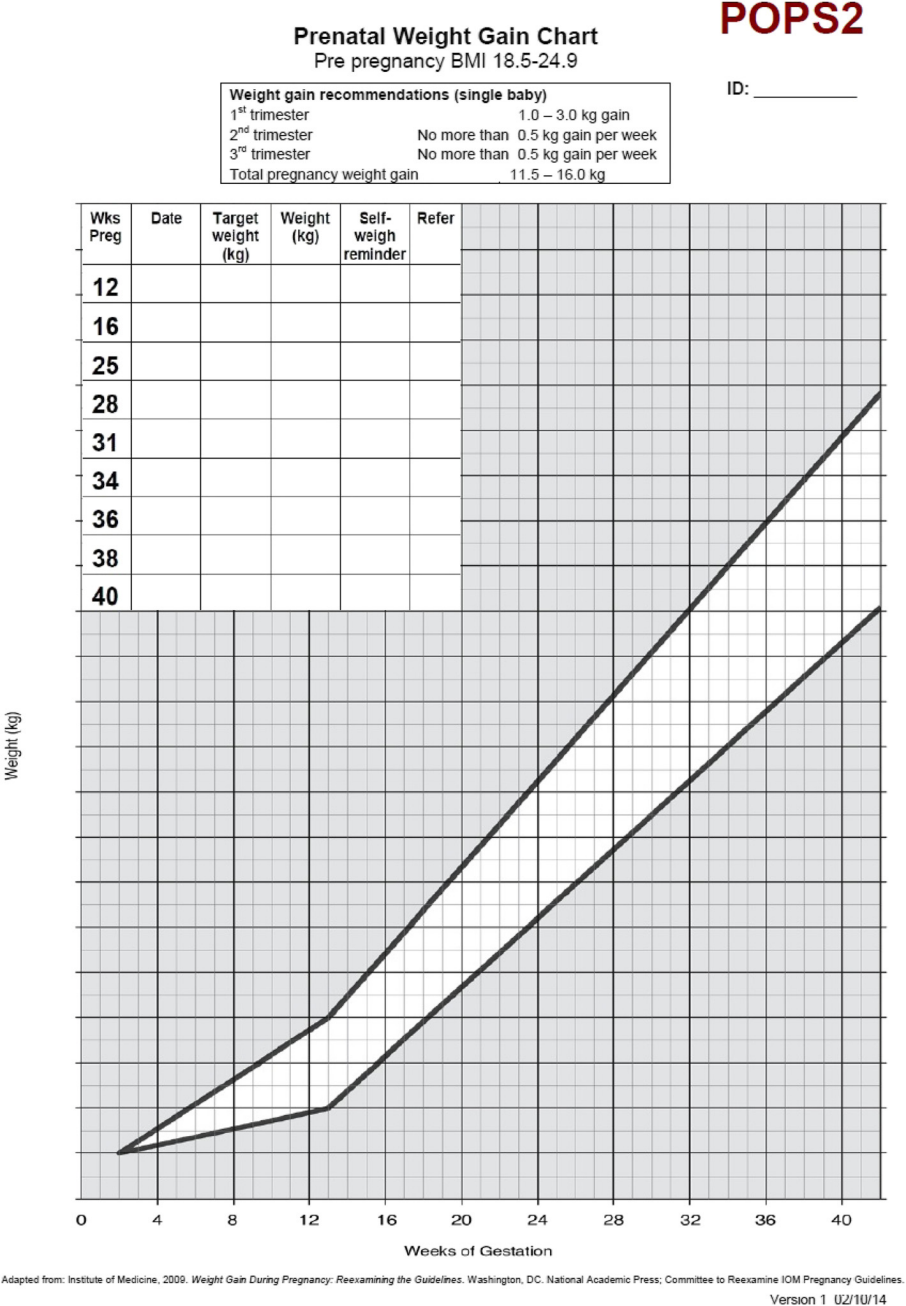
Example weight gain chart

**Fig. 2:**
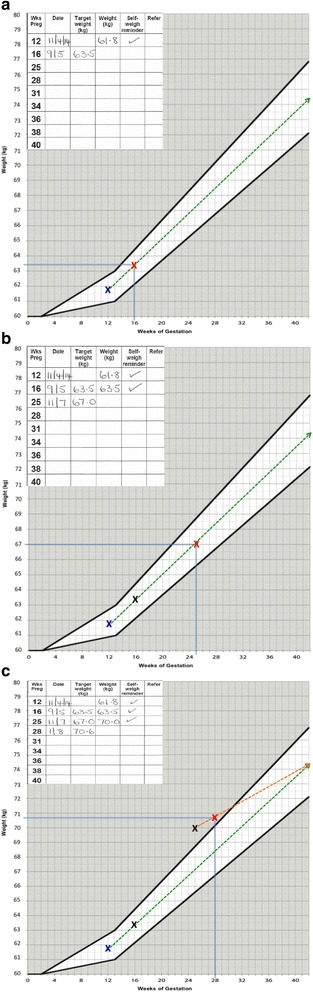
**a**: Example of how to set weight gain limits for antenatal appointments. The woman is recruited at 12 weeks gestation and her weight is plotted on the chart for this week of pregnancy. The woman is advised that her weight should follow the dotted line drawn through the ideal weight gain zone on the chart (unshaded area). The woman is due to be seen again by her midwife at 16 weeks gestation therefore the midwife draws a vertical line at 16 weeks gestation to meet the dashed line in the unshaded ideal weight gain zone to ascertain what the maximum weight target should be for 16 weeks gestation. In this example the woman is advised by her midwife that ideally her weight should be no more than 63.5 kg at 16 weeks gestation. The midwife repeats the procedure at each antenatal appointment. **b**: Example of how to set weight gain limits for antenatal appointments. At 16 weeks of pregnancy the midwife weighed the woman and plotted her weight on the chart. In this example the woman weighed 63.5 is 16 weeks gestation which was the maximum weight limit set at her previous appointment at 12 weeks gestation. The midwife then set the maximum weight target for the next antenatal appointments which was scheduled for 25 weeks gestation. The midwife draws a vertical line at 25 weeks of gestation to meet the dashed line in the unshaded ideal weight gain zone to ascertain what the maximum weight target should be for 25 weeks gestation. In this example the woman should ideally weigh no more than 67 kg at 16 weeks gestation. **c**: How to set and adjust maximum weight targets if women gain too much weight. At 25 weeks gestation the midwife weighed the woman and plotted her weight, which was 70 kg. This was above the maximum weight target set at the previous appointment at 16 weeks gestation. The midwife therefore redraws the ideal weight trajectory line starting from the plotted weight at 25 weeks gestation to the central point in the unshaded weight zone until 42 weeks gestation. The midwife uses this new line to set the maximum weight target for the next antenatal appointment scheduled for 28 weeks gestation. The midwife draws a vertical line at 28 weeks of gestation to meet the dashed line in the unshaded ideal weight gain zone to ascertain what the maximum weight target should be for 28 weeks gestation. The midwife advised the woman that her maximum weight target for 28 weeks of pregnancy was 70.6 kg

**Fig. 3:**
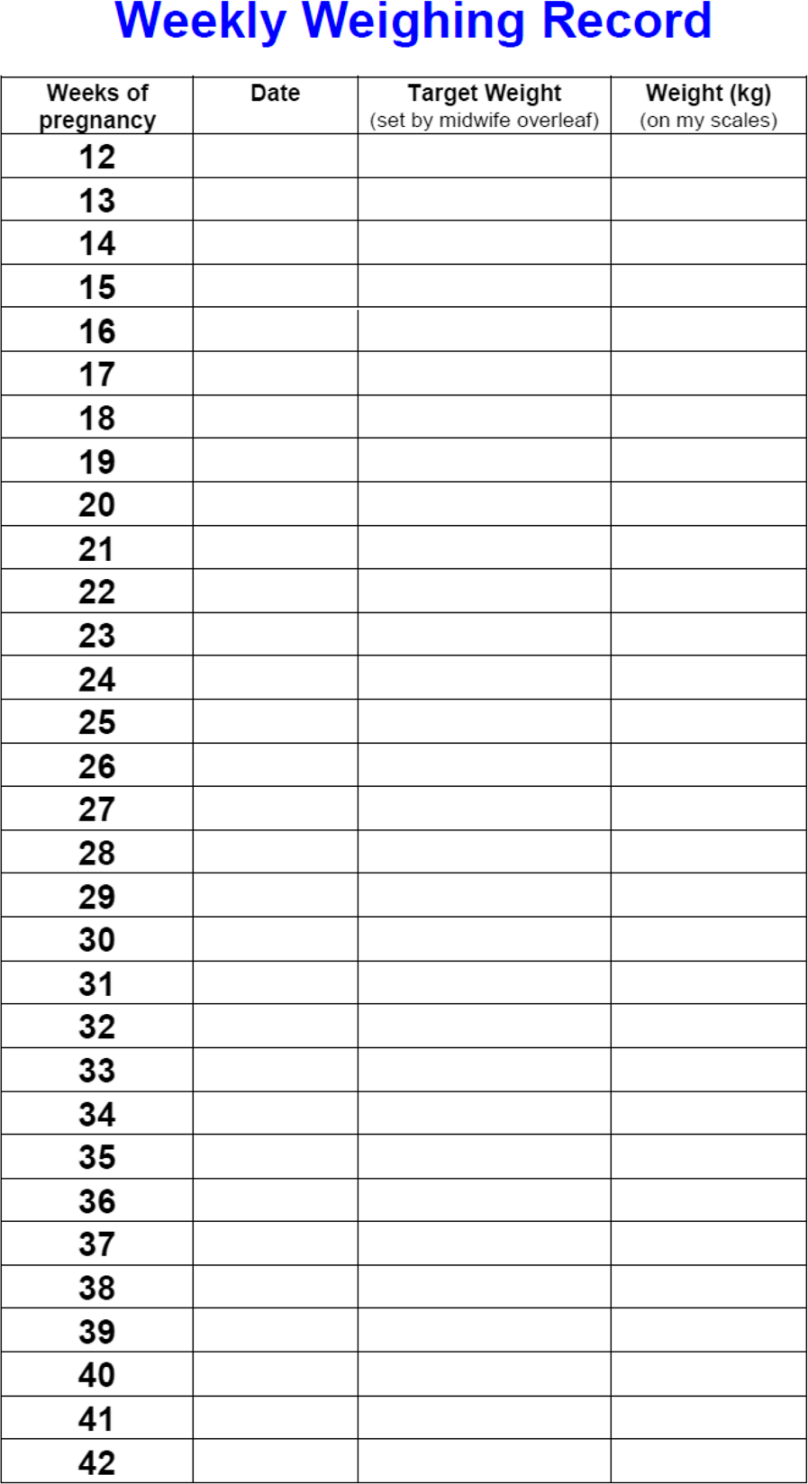
Weight record card

In preparation for this phase III trial we conducted a feasibility RCT with 76 pregnant women randomised to usual antenatal care only or usual antenatal care plus an intervention involving community midwives setting targets for healthy weight gain, regular weighing and providing feedback on progress. The intervention was delivered by eight community midwives who weighed women in their care, plotted their weight on a personalised BMI specific IOM weight gain chart, gave feedback on progress according to the weight chart (see Fig. 1) and set a maximum weight gain limit for women at each antenatal appointment. The weight target was adjusted at each appointment if women had exceeded their weight gain target set at the previous appointment (Fig. 2a–c). Targets were always for weight gain even if maximum recommended weight gain had already been exceeded. Women were given a weight record chart and asked to weigh themselves weekly between appointments to check their own weight gain (see Fig. 3).

We developed a training module for midwives to deliver the intervention. We also looked for evidence of intervention contamination in the usual care group. We obtained feedback through semi structured interviews from midwives about their experiences of implementing the intervention within routine care. More detail about the feasibility study and the results has been published elsewhere [50]. In summary, the intervention proved feasible to deliver and was considered acceptable by pregnant women and community midwives. Data showed a modest but promising difference favouring the intervention group in the percentage of women exceeding the IOM recommended weight gain for their early BMI category (23.5 % versus 29.4 %) at 38 weeks of pregnancy. The trial was not planned to be large enough to estimate the difference in the proportion achieving healthy weight gain as this is the purpose of a definitive effectiveness trial. There was no evidence the intervention caused undue anxiety in the intervention group relative to usual care. In the usual care group we found no evidence of intervention contamination where midwives gave the intervention to this group. The intervention group reported higher total physical activity scores at 38 weeks of pregnancy than usual care. The intervention group also reported participation in substantially more vigorous intensity physical activity (double the rate) than usual care at 38 weeks gestation.

### Views of community midwives: feasibility study

Most midwives commented that they had had some initial apprehension about delivering the intervention because it was something new, but that over time it became second nature. All the midwives felt it was feasible to deliver the intervention within the context of routine antenatal care, taking on average about one to two minutes per appointment, which was not perceived as adding substantially to their workload. Midwives liked the intervention because it was simple to do. Midwives felt the weight gain chart provided them with a legitimate opportunity or ‘excuse’ to raise the topic of gestational weight gain.

### Views of pregnant women: feasibility study

Twelve intervention participants were interviewed 6–8 weeks postnatally. Women reported the main reason that they liked the intervention was that it might help them avoid gaining too much weight during pregnancy. Women particularly liked being weighed at each appointment because it provided on-going motivation to consider their lifestyle choices. Women felt the intervention was useful in helping them to be more aware and vigilant about their eating and physical activity and they appreciated having a target weight set by the midwife. Women did not have any concerns about discussing their weight with their midwife and the majority felt that regular weighing during pregnancy was important and worthwhile and some had recommended self-weighing to others. Most of the women interviewed weighed themselves each week between antenatal appointments, but only a few recorded this on their record chart, highlighting that additional strategies to encourage this behaviour will be needed in the phase III trial.

## Methods

### Primary objective

- To assess the effectiveness of a brief intervention to encourage healthy weight gain during pregnancy.

### Secondary objectives

- To assess the difference in weight gain per weeks of pregnancy between the groups.
- To compare the difference in change in total weight gain during pregnancy between the groups.
- To compare the difference in changes in psychological health, physical activity and diet quality between the groups.
- To compare the occurrence rates of common complications of pregnancy (e.g. c-section, hypertension in pregnancy and gestational diabetes) between the groups.

### Study design

**Fig. 4:**
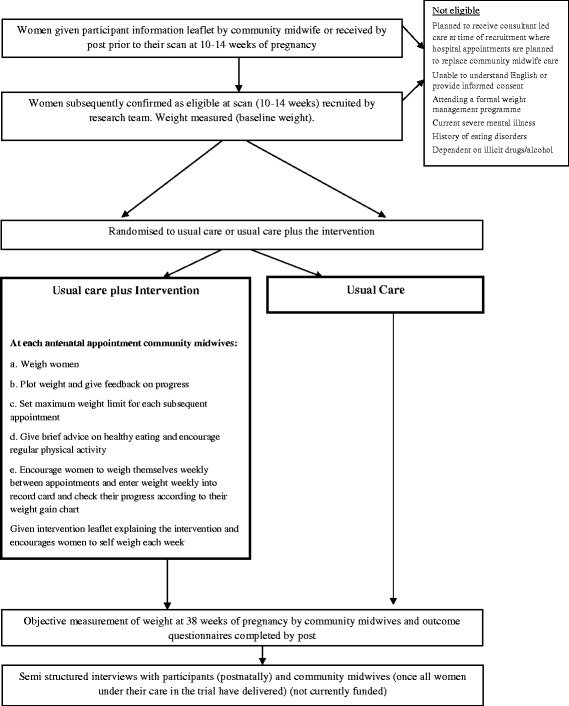
Study flow diagram

This study is a two group RCT with participants allocated to receive usual antenatal care only or usual antenatal care plus the intervention. Participants will be individually randomised to the trial groups. See Fig. 4 for trial flow

### Outcome measures

The primary outcome is the proportion of participants per group who exceed the IOM guidelines for gestational weight gain according to their early pregnancy BMI category from baseline to 38 weeks of pregnancy. Secondary outcomes are the difference in weight gain per weeks of pregnancy between the groups**,** difference in total weight from baseline to 38 weeks of pregnancy between the groups and difference in changes in psychological health, physical activity and diet quality between the groups from baseline to 38 weeks of pregnancy.

### Identification and recruitment of pregnant women

Women will receive the participant information leaflet from their community midwife at their first antenatal appointment or by post. Women will be advised to read the leaflet and that they may be approached to participate in the study after they have had their dating scan at 10 –14 weeks of pregnancy.

### Eligible women who do not consent

The researcher at the scan clinics will record the number of women each day who are eligible but do not provide consent to participate when approached. With the verbal consent of the woman the researcher making the approach will log the BMI status, ethnicity and age of eligible women approached who do not consent to participate in the study.

### Recruitment

This trial is multisite with participants recruited from four maternity centres in the UK; Birmingham Women’s NHS Foundation Trust, The Dudley Group NHS Foundation Trust, Oxford University Hospitals NHS Trust (OUH) and South Warwickshire NHS Foundation Trust where there are 7670, 4610, 6500 and 3456 births per year respectively (total yearly births 22,236). Of these 97 % (*n* = 21,568) will be of either healthy weight, overweight or obese at booking, and of these we conservatively expect 55 % to be confirmed as receiving community midwife led care (*n* = 12,230) and fully eligible, demonstrating there is a very large population available from which we can recruit. We plan to enrol 610 pregnant women.

### Sample size

We will recruit 610 participants, with 305 randomised to each group.

### Planned inclusion criteria

- Aged 18 years or more
- Between 10^+0^ and 14^+6^ weeks of pregnancy
- Singleton pregnancy with a BMI ≥18.5 kg/m^2^ at the time of recruitment planned to receive community midwife led care or shared care where it is anticipated any consultant appointments will be in addition to community midwife appointments, at the time of recruitment. This criteria is included because it is important that women enrolled into the study who are randomised to the intervention group receive all the intervention contacts with their community midwife.

### Planned exclusion criteria

- Women referred for consultant led care where it is anticipated that midwife appointments will be substituted by consultant appointments
- Unable to understand English or provide informed consent
- Women who are attending a weight management programme (i.e. Slimming World Whilst Pregnant)
- Current severe mental illness, known history of eating disorders or dependent on illicit drugs or alcohol

### Randomisation and blinding

The telephone randomisation service at the Primary Care Clinical Research Trials Unit (PCCRTU) will be used to randomly allocate participants to trial groups. The randomisation list will be developed by a statistician within the Trials Unit using NQuery Advisor. Participants will be individually randomised to usual care plus intervention or usual care only on a 1:1 basis using random permuted blocks of random size. The list will be stratified by BMI category at recruitment (healthy weight, overweight, obese) and maternity site (Birmingham, Oxford, Dudley & Warwick). The primary outcome is measured by community midwives at routine 38 week pregnancy appointments, therefore they will not be blinded to group allocation but we have no reason to believe midwives will not record these data accurately. The trial statistician will remain blinded to group allocation until analysis is completed.

### Intervention

No official clinical guidelines for weight gain during pregnancy exist in the UK or in many other countries (e.g. Australia, Ireland, Netherlands, New Zealand & Sweden) and there are no global maternal weight gain guidelines [23]. The only available guidelines for weight gain during pregnancy were developed by the IOM in the USA [4]. While we are confident the IOM guidelines are broadly appropriate for a UK population where this study is based, the principles of our intervention are such that it could be used with any UK or country specific guidelines that might be subsequently adopted. The intervention will supplement usual antenatal care. Community midwives will be asked to weigh women at each antenatal appointment and plot their weight on an IOM weight gain chart appropriate to their BMI at recruitment. The chart will be attached to the woman’s hand held pregnancy notes and will outline a maximum weight gain limit for each subsequent appointment. Women will be given brief feedback on their progress emphasising the importance of healthy weight gain. The intervention will actively engage women in self monitoring of their weight by them recording their weekly weight on a record chart and encouraging them to use their weight chart to reflect on what their maximum weight limit is for each week of pregnancy and to take action if required.

#### Regular weighing and weight gain charts

All study community midwives will be issued with calibrated light weight portable weighing scales and instructed to use these for this study. Women will be weighed by their midwife at each antenatal appointment. After 12 weeks of pregnancy in usual antenatal care there are up to eight consultations or opportunities for the intervention to take place. The explicit behavioural goal of the intervention will be for women’s weight gain to follow the trajectory of the midpoint line of the healthy weight gain zone of their IOM chart (Fig. 2a–c) to prevent excessive weight gain. In line with NICE guidance the goal will always be for weight gain, and never weight loss. The weight gain charts are personalised according to women’s weight and BMI category (healthy weight, overweight, obese) at recruitment.

Many women do not know their pre pregnancy weight therefore when the weight gain chart is prepared and customised at recruitment it will be assumed that women have gained average weight at recruitment in line with weeks of gestation according to their BMI category. BMI and therefore the BMI category weight chart used will be based on participants’ BMI when measured at recruitment.

#### Setting the parameters for gestational weight gain

The intervention will support women to stay on course for healthy overall weight gain by setting a maximum weight gain limit for each subsequent antenatal appointment that encourages women to stay within the two threshold lines on their weight chart. As used in the feasibility trial we will teach midwives a simple method for adjusting women’s maximum weight gain limit that aims to move women’s weight gain back to the healthy zone, but safely and slowly (see Fig. 2a–c). Women whose weight gain is within the appropriate range on the chart will be told they are gaining the ideal amount of weight and encouraged to maintain a healthy lifestyle. Women gaining too much weight will be encouraged to eat a healthy diet and restrict their intake of high fat and sugary foods and drinks and to participate in regular physical activity (walking). Women who are gaining too little weight will be referred by their midwife to the appropriate health professional in line with local practice. As we do not want women to lose weight at any stage in their pregnancy it may not be possible to meet the final target if early weight gain is excessive.

#### Advice about gestational weight gain and lifestyle

As part of the feedback from the weight gain charts, midwives will give messages about the importance of preventing excessive weight gain during pregnancy, address misconceptions about nutritional needs during pregnancy (e.g. “*eating for two*”, “*weight gain does not matter when you are pregnan*t*”*, “*you shouldn’t exercise when you are pregnant*”) and offer usual advice about healthy eating and exercise in pregnancy. This is intended as a brief intervention that could be implemented into routine care, so midwives will not be expected to engage women in detailed lifestyle counselling about how changes to diet and physical activity might be implemented; the focus will be on giving brief feedback and advice. Women will be encouraged to accumulate 30 min of moderate intensity physical activity (walking) each day in line with current recommendations [51, 52]. Brief advice about healthy eating will include portion control, decreasing energy dense foods, limiting sugar-sweetened beverages, increasing and/or maintaining consumption of fruit and vegetables. If women are already following a healthy lifestyle midwives will encourage continuation of these health behaviours and emphasise why it is important to do so during pregnancy. The focus of the intervention is on setting targets, providing feedback, and encouraging self-monitoring. It is not on detailed nutritional and exercise counselling.

#### Self weighing and monitoring of weight

Women will be asked to monitor weight by weighing themselves weekly and will be reminded to do so by their community midwife at each appointment. They will assess their progress towards the maximum weight gain limit set by their midwife on the weight gain chart; this will provide women with a visual picture of their weight gain progress to date and a prompt to take action if they are not on target. In addition women will be asked to write down their weight on their weight record card and set themselves a reminder to do so between appointments with the midwife (Fig. 3). Collectively these tasks are important for several reasons. We learnt from our feasibility trial that we needed to place greater emphasis on women self managing their weight between appointments and have established strategies to help achieve this. There are gaps of several weeks between some antenatal appointments (e.g. between 16–24 weeks of pregnancy) so it is important for women to be aware of their weight gain during these intervals, so that lapses can be identified early and correction implemented. The intervention seeks to encourage women to work in partnership with their midwife rather than relying entirely on the midwife for feedback before taking action. We also want women to become familiar and comfortable with using their weight gain chart to understand what their weight should be for each week of pregnancy.

Women who do not have scales at home will be given some to use at recruitment. At baseline women will be advised to check their weight when they get home on their own scales to assess whether their home scales are measuring the same weight as recorded by their midwife. Women who have scales that do not record weight accurately (within 0. 5 kg of the measured weight at recruitment) will be asked to contact the study coordinating centre which will issue new scales. Women will be encouraged to only weigh themselves once per week. Women will be encouraged to work out when and where they plan to weigh themselves and write down their weight on the record card. Helping women form implementation intentions should increase adherence.

#### Intervention leaflet

Women randomised to the intervention will be given a brief leaflet outlining the key principles as described here and it will explain to women why it is important to weigh themselves each week and check their weekly maximum weight gain limit. Examples of how women might implement weekly weighing (implementation intentions) will also be included in the leaflet.

#### Training community midwives to deliver the intervention

The training module was developed for the feasibility trial and modified based on feedback from community midwives. It takes 60 min to deliver and is designed for group training, with information on study eligibility criteria, recruitment procedures and the importance of adhering to protocol. It describes the intervention, including information on the consequences of weight gain during pregnancy, instructions about how to weigh and plot weight on the chart, how to give feedback on the weight gain chart and example messages, set weight gain limits using the charts, and examples of educational and motivational messages about gestational weight gain, diet and physical activity during pregnancy. Midwives will practice completing the weight gain charts using prepared case studies. The training module will include example cases demonstrating how the intervention works in practice, the kind of questions likely to be raised by women and ideas of how to deal with them.

### Intervention implementation and fidelity

Early in the study we will select a random sample of 50 charts from different midwives across study sites to be checked by the research team for completion and accuracy when women are 20 weeks pregnant. This will provide an indication if additional training of midwives is required. To provide a measure of intervention implementation in the intervention group as a summary on trial completion we will obtain participants’ weight gain charts and weight record cards and assess accuracy. From the weight gain chart we will check whether weight had been measured, plotted and recorded by community midwives and if maximum weight gain limits were calculated and recorded correctly at each antenatal appointment. We will assess how frequently midwives set maximum weight targets and how often they encouraged women to weigh themselves weekly. We will assess how often women in the intervention group completed their weekly weight record to measure their engagement with the intervention. We will assess whether midwives delivered advice according to the protocol in the intervention group and did not contaminate the trial in the usual care group by asking women in a questionnaire about the advice given by their community midwife about weight control, eating, and physical activity.

### Usual care

The usual care group will receive standard maternity care according to local health care provision and no other intervention. NICE [42, 53] state that women should be given information on diet and exercise early in pregnancy. NICE [42, 53] do not currently recommend that midwives routinely weigh women or give information about optimal weight gain during pregnancy. This is not a trial about giving lifestyle advice so midwives will not be asked to refrain from offering usual advice about diet and exercise early in pregnancy. This trial is testing the addition to usual care of an intervention where midwives set targets for healthy weight gain, weigh women and encourage women to monitor their weight to ensure they stay on target, and give feedback on every visit for antenatal care.

### Descriptive data

Data will be collected about participants’ demographic details, smoking status and weight management history at recruitment.

### Data, outcomes and process measures

See Table 1 for a comprehensive list of the outcomes and process measures in the study.

**Table 1:** Data, outcomes and process measures

Recruitment measures		
Eligible participants not consented		
Number declined at booking scan		
Number of participants randomised		
	Usual care group	Intervention group
Process measures		X
Accuracy and completion rates of weight chart by midwives		X
How frequently did midwives encourage women to weight themselves weekly		X
Frequency of regular weighing by participants as recorded on weight record cards		X
Outcomes		
Weight	X	X
Depression and anxiety (HADS) [55]	X	X
Physical activity (Physical activity in pregnancy questionnaire [56]	X	X
Diet quality (Southampton food frequency questionnaire) [57]	X	X
Questions about diet and physical activity advice midwives have given during pregnancy	X	X
Maternal and neonatal complications (obtained from electronic hospital records)	X	X

### Baseline assessment

All trial women will be weighed by the research team using calibrated scales and have their height measured with excess clothing (i.e. shoes and coats) removed. The study baseline questionnaires will also be completed measuring anxiety and depression, physical activity and diet quality.

### Follow up

We rehearsed several strategies in our feasibility trial for ensuring high follow up rates for our primary outcome in this trial (>90 % obtained in our feasibility trial). Pregnant women are routinely seen by their midwife at 38 weeks of pregnancy and they will be weighed at this appointment for the purpose of this study. At recruitment all women will have a sticker inserted into their hand held pregnancy notes on the page where midwives normally record routine medical information; weight and weeks of pregnancy will be recorded on this sticker. Women who do not attend their 38 week appointment will be contacted and alternative arrangements for them to be weighed will be made. We have chosen 38 weeks of pregnancy as the primary outcome because this time represents nearly the end of pregnancy and only 7–10 % of women deliver before this gestation [54], which leads to a low rate of unavoidable loss to follow-up. As women will see their community midwife weekly until the end of pregnancy after 38 weeks, this provides several more opportunities to catch up with women after 38 weeks if women miss the 38 week appointment or the midwife does not weigh women. As a consequence, we believe loss to follow up will be low. At 38 weeks, the study team will post a questionnaire to women to assess depression and anxiety, physical activity and diet quality. The team will send one postal reminder to non responders.

### Post study data collection

The weight gain charts will be retrieved from the pregnancy notes after women have had their baby. We do not expect to have sufficient power within this trial to detect statistically significant differences in maternal and neonatal complications but these data will be obtained from medical records at each hospital and described in the trial reports to contribute to future meta analyses. The following information on pregnancy will be collected mode of delivery, inpatient days for the mother, maternal ICU admission, preeclampsia, pregnancy induced hypertension, gestational diabetes, maternal sepsis, preterm delivery, miscarriage, stillbirth, shoulder dystocia, treatment for jaundice, admission to the neonatal intensive care unit (duration of stay if relevant and inpatient days), neonatal death and 1 and 5 min Apgar score and neonatal sepsis. Data on infant birth weight and gestational age at delivery will also be collected from hospital electronic records.

### Adverse and serious adverse events requiring hospitalisation

There are several reasons to believe the intervention is safe and will not cause adverse events. We are not testing a dangerous intervention; the treatment consists of regular weighing and feedback as part of routine antenatal care, along with self monitoring of weight at home, none of which seem likely to create harm. Therefore, we will not monitor the occurrence of all adverse events by trial group as it will be burdensome for midwives and participants. We will only report unexpected serious adverse events and deaths to the research ethics committee. Expected serious adverse events (that will not be reported to the ethics committee) are defined as preeclampsia, gestational diabetes, stillbirth, neonatal death, termination due to abnormality, gestational hypertension, cerebrovascular accident, myocardial infarction, pre term delivery, termination, miscarriage, severe mental health illness, antepartum haemorrhage, thromboembolism, maternal sepsis, maternal admission to intensive care, hysterectomy, visceral injury, neonatal admission to the special care baby unit, neonatal infection, neonatal respiratory distress.

### Sample size

A total of 610 (305 per group) is sufficient to detect a 15 percentage points difference (45 % versus 60 %) [4–7, 39] in the proportion who, at 38 weeks of pregnancy, exceed the IOM guideline for gestational weight gain according to their baseline pregnancy BMI in the intervention group compared to usual care (90 % power, 5 % significance level). Previous prevention trials [31, 41], have reported between 10–25 % points difference between the proportion in intervention and control groups that exceed the IOM cut-offs for gestational weight gain, depending on intervention intensity and the population, we have been cautious in expecting 15 % points difference. The sample size includes allowance for 20 % loss to follow up at 38 weeks of pregnancy. 610 participants would also be sufficient to detect 1.6 kg difference in mean weight at 38 weeks of pregnancy (SD weight change of 5.5 kg from our pilot RCT [50], 90 % power, 5 % significance level).

### Statistical analysis

Baseline characteristics including age, social class, ethnicity and parity will be presented by randomisation group using summary statistics. The primary analysis will focus on the proportion of women who exceed the IOM guidelines for gestational weight gain at ~38 weeks of pregnancy (primary outcome). The difference in these proportions between the groups (and odds ratio) will be calculated along with associated confidence interval using mixed effects logistic regression modelling. The analysis will adjust for BMI category (healthy weight, overweight, obese) and site (Birmingham, Dudley, Oxford, Warwick) as fixed effects and midwife as a random effect. Missing weight data for births occurring after 37 weeks (excluding pregnancy loss), will be imputed using multiple imputation. Linear mixed modelling will be used to compare the secondary outcomes of weight gain per weeks of pregnancy, depression, anxiety, physical activity and diet recorded at ~38 weeks of pregnancy. Mother and baby pregnancy complication rates will be summarised by group. In a planned but exploratory subgroup analysis, BMI categories (healthy weight, overweight, obese) will be compared for the primary outcome by its inclusion as an interaction term (intervention by BMI category) in the modelling. There are no other planned subgroup analyses. A sensitivity analysis of the primary outcome will be performed without imputing missing weights. Treatment effects, 95 % confidence intervals, and two-sided p values will be reported for all comparisons. Analyses will be undertaken using an intention to treat approach. Full details of analysis will be pre-specified in a separate statistical analysis plan.

### Trial steering committee and data monitoring committee

A trial steering committee (TSC) will be convened to provide overall supervision of the trial and ensure its conduct is in accordance with the principles of Good Clinical Practice and the relevant regulations. The TSC will agree the trial protocol and any protocol amendments. Furthermore the TSC will provide advice to the investigators on all aspects of the trial. We do not propose that a data monitoring and ethics committee would be useful as this is an unblinded study with no substantial risk and no early termination rules.

### Ethical approval

The Chief Investigator has obtained favourable ethical approval for the study from the South Birmingham NRES Committee (14/WM/1134). The study will be conducted in accordance with the recommendations for physicians involved in research on human subjects adopted by the 18^th^ World Medical Assembly, Helsinki 1964 and later revisions. The University of Birmingham will act as the sponsor. All the hospitals involved in this research have ethically reviewed and approved the protocol.

## Discussion

This study will evaluate the effectiveness of an intervention to prevent excessive weight gain. This study has design strengths as well as some limitations. We have followed best practice in designing the randomisation procedures and implementing concealment of allocation. In common with practically all trials of behavioural interventions, it was impossible to blind either participants or midwives to which group they are in. However, it is unlikely that knowledge of group allocation will particularly affect other maternity care offered to women that might prevent excessive weight gain. Midwives rarely engage on this topic in routine care and part of the impact of the intervention of weighing women, giving feedback, and setting maximum weight gain limits is to prompt discussion of the importance of healthy weight gain and how to achieve it in the intervention group. In this trial, our primary outcome is assessed by midwives who are not blind to treatment allocation. In deciding to do this, we faced a trade-off between participant and midwife burden and achieving high rates of follow-up on the one hand and between blinded follow-up on the other. We believe that women are highly motivated to attend maternity care and are much more likely to be motivated and able to attend for this than they would be for blinded follow-up for weighing by a researcher. As weight is objective, we think the risk of bias from this compromise is low and less than the risk of attrition bias that we judged more likely trying to implement blinded follow-up, particularly for the control group for whom being weighed may not seem that important. By declaring our outcomes in advance and writing a detailed statistical analysis plan we have protected against selective reporting bias.

A lack of high quality trials has hindered the development of recommendations for clinical practice in the UK and many other countries. The proposed trial will test an intervention that can be delivered at scale as part of routine antenatal care incurring minimal additional costs, using the professional expertise of community midwives but not requiring specialist weight management skills. Given that it is impossible to predict who will gain excessive weight, we need interventions that need to be applied to all women, implying that they must be low intensity and inexpensive. If an intervention such as this was shown to be effective, it seems likely that it could immediately become part of routine maternity care at a small cost, without increasing substantially antenatal appointment length. Other than our pilot RCT, this is the first trial to investigate the benefits of setting weight limits and regular weighing by community midwives.

## Abbreviations

APGAR: Appearance, Pulse, Grimace, Activity, and Respiration
BMI: Body Mass Index
GCP: Good Clinical Practice
IOM: Institute of Medicine
kg: kilograms
NICE: National Institute for Health and Care Excellence
NRES: National Research Ethics Service
RCT: Randomised Controlled Trial
SD: Standard Deviation
TSC: Trial Steering Committee
UK: United Kingdom
USA: United States of America
